# Supplementary material for: Investigating the influence of physiologically relevant hydrostatic pressure on CHO cell batch culture
Source: Sci Rep. 2021 Jan 8;11:162. doi: 10.1038/s41598-020-80576-8 (PMC7794228; doi:10.1038/s41598-020-80576-8)
Supplement: Supplementary file 1 — Supplementary Information 1. [file 41598_2020_80576_MOESM1_ESM.zip › S1/IgG/Feb17 60 90 standard/standard/20ugml volumn.pdf]

===

Acq. Operator : SYSTEM Seq. Line : 13
Sample Operator : SYSTEM
Acq. Instrument : HPLC-0039 Location : 6
Injection Date : 2/17/2019 6:21:13 PM Inj : 1
Inj Volume : 100.000 µl
Different Inj Volume from Sample Entry! Actual Inj Volume : 20.000 µl
Sequence File : C:\Users\Public\Documents\ChemStation\1\Data\TK_CHO_IgG 2019-02-17 17-28-56\TK_CHO_IgG.S
Method : C:\Users\Public\Documents\ChemStation\1\Data\TK_CHO_IgG 2019-02-17 17-28-56\CHO_IgG_LC.M (Sequence Method)
Last changed : 2/8/2019 3:17:23 PM by SYSTEM

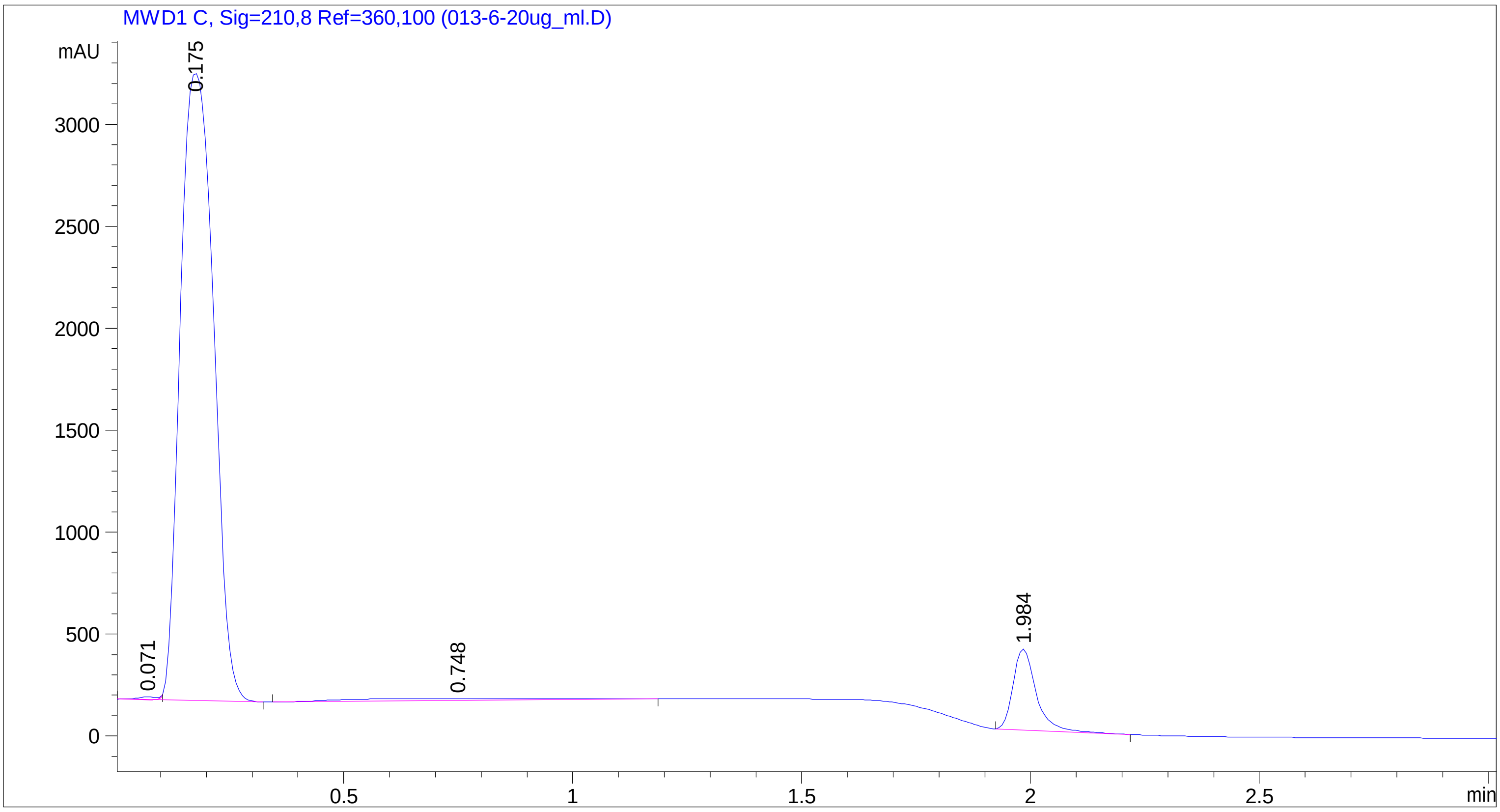

===

Area Percent Report

===

Sorted By : Signal
Multiplier : 1.0000
Dilution : 1.0000
Use Multiplier & Dilution Factor with ISTDs

Signal 1: MWD1 C, Sig=210,8 Ref=360,100

Peak #	RetTime [min]	Type	Width [min]	Area [mAU*s]	Height [mAU]	Area %
1	0.071	BV E	0.0454	38.05970	13.57104	0.2203
2	0.175	VB R	0.0830	1.55557e4	3078.08398	90.0383
3	0.748	BB	0.4089	302.31720	8.97784	1.7498
4	1.984	BBA	0.0527	1380.68347	396.04837	7.9916

Totals : 1.72768e4 3496.68123

==
*** End of Report ***
